# Redox double-switch cancer theranostics through Pt(iv) functionalised manganese dioxide nanostructures†

**DOI:** 10.1039/d3nr00076a

**Published:** 2023-06-13

**Authors:** Beatriz Brito, Maria Rosaria Ruggiero, Thomas W. Price, Milene da Costa Silva, Núria Genicio, Annah J. Wilson, Olga Tyurina, Veronika Rosecker, Thomas R. Eykyn, Manuel Bañobre-López, Graeme J. Stasiuk, Juan Gallo

**Affiliations:** a Department of Imaging Chemistry and Biology, School of Biomedical Engineering and Imaging Sciences, King's College London Strand WC2R 2LS London UK graeme.stasiuk@kcl.ac.uk; b School of Life Sciences, Faculty of Health Sciences, University of Hull Cottingham Road HU6 7RX Hull UK; c Advanced Magnetic Theranostic Nanostructures Lab, International Iberian Nanotechnology Laboratory Av. Mestre José Veiga 4715-330 Braga Portugal juan.gallo@inl.int manuel.banobre@inl.int

## Abstract

Manganese dioxide (MnO_2_)-based nanostructures have emerged as promising tumour microenvironment (TME) responsive platforms. Herein, we used a one-pot reaction to prepare MnO_2_ nanostructures with Pt(iv) prodrugs as redox- (and thus TME-) responsive theranostics for cancer therapy, in which the Pt(iv) complexes act as prodrugs of cisplatin (Pt(ii)), a clinical chemotherapeutic drug. The cytotoxicity of these MnO_2_–Pt(iv) probes was evaluated in two and three dimensional (2D and 3D) A549 cell models and found to be as effective as active drug cisplatin in 3D models. Moreover, MnO_2_–Pt(iv) nanoparticles exhibited strong off/ON magnetic resonance (MR) contrast in response to reducing agents, with the longitudinal relaxivity (*r*_1_) increasing 136-fold upon treatment with ascorbic acid. This off/ON MR switch was also observed in (2D and 3D) cells *in vitro*. *In vivo* MRI experiments revealed that the nanostructures induce a strong and long-lasting *T*_1_ signal enhancement upon intratumoral injection in A549 tumour-bearing mice. These results show the potential of MnO_2_–Pt(iv) NPs as redox responsive MR theranostics for cancer therapy.

## Abstract 2

A new redox-responsive double-switch cancer theranostic was developed based on Mn nanostructures and a Pt prodrug. The ability of these probes to respond to altered redox states was validated in 2/3D cell models and in a preclinical mouse model.

## Graphical abstract

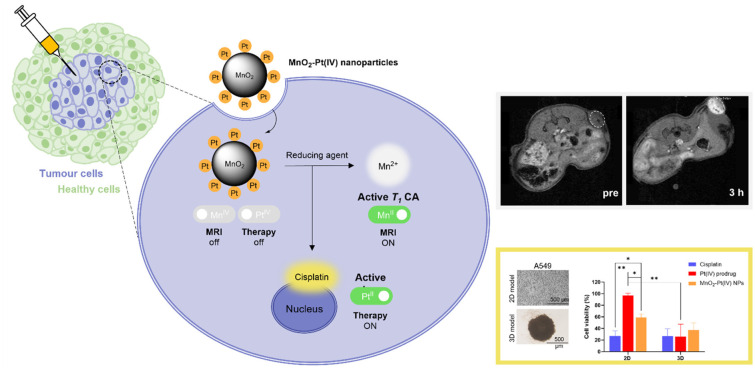

## Introduction

Conventional cancer therapy involving surgical resection, chemotherapy, radiotherapy and their combination has significantly improved cancer survivability. However, severe side effects and resistance phenomena particularly in the case of chemotherapy, have prompted the development of new approaches, for example, immunotherapy has joined the aforementioned treatments as a pillar of cancer management.

Theranostics is an alternative approach that enables simultaneous treatment and monitoring with fewer off-target effects by combining an imaging moiety with a therapeutic functionality in a single entity. This can offer synergistic advantages in cancer therapy when compared to standard treatment alone.^1^ Theranostic agents can provide feedback on drug distribution to target sites and enable more efficient monitoring of the response to therapy.^2,3^ Smart or responsive theranostics are platforms in which the therapeutic and/or imaging components undergo structural or physicochemical alterations to release/activate the active component – a drug, a contrast agent or both – in response to a certain stimulus after reaching the target tissue. The triggers for these imaging and/or therapeutic switches can be exogenous (*e.g.* light, magnetic fields, ultrasounds) or endogenous (*e.g.* redox environment, pH, enzymes).^4,5^ Endogenous mechanisms aim to exploit the differences between diseased and healthy tissues. The redox potential of neoplastic tissues is distinct from healthy tissues. Indeed, glutathione (GSH), which plays a major role in the regulation of the redox status of cells, is overexpressed in cancer tissues: there is a 4 times higher concentration of GSH in a tumour microenvironment *vs.* healthy tissues,^6–9^ and there are 100–1000 times higher GSH concentrations in tumour cells cytoplasm *vs.* blood and extracellular fluids.^10^ Responsive theranostic agents can be designed with bonds that are sensitive to a reducing environment, meaning their therapeutic and/or imaging functions are preferentially activated in GSH-rich environments, such as the tumour microenvironment (TME) or inside cancer cells.^11–15^ Manganese dioxide (MnO_2_) nanostructures have recently been proposed as redox-responsive agents and act as responsive *T*_1_ contrast agents for MRI.^16,17^ In MnO_2_ nanoparticles, manganese presents a 4+ oxidation state and shows weak paramagnetism, which translates into a negligible effect on the relaxation rates of water protons measured by MR. However, in the presence of biologically relevant concentrations of GSH or other redox active species,^18^ MnO_2_ nanostructures are degraded/reduced to free aqueous Mn(ii) and O_2_.^19^ Mn(ii) (2+ oxidation state) is strongly paramagnetic and its effect on the MR signal is greatly enhanced. Previous work has reported a 140-fold *T*_1_ signal enhancement upon treatment of MnO_2_ nanoparticles with reducing agents.^16^ MnO_2_ nanoparticles therefore have redox-responsive off/ON MR behaviour, which can be exploited for the detection of tumours.

Moreover, it has been described that the reduction of MnO_2_-based nanomaterials in the TME allows for the increase of local O_2_ concentration^20,21^ and decrease of antioxidant concentration in tumours,^14,22–24^ meaning that these materials have high potential as therapeutic platforms. These anticancer properties have been enhanced by combining MnO_2_-based particles with different functional agents, from cisplatin,^25,26^ doxorubicin^27,28^ or enzymes to porphyrins.^29,30^ Indeed, MnO_2_-based nanomaterials have found applications in a wide variety of different therapeutic modalities,^24^ including chemotherapy,^17,26^ photodynamic therapy,^21,31^ chemodynamic therapy,^32,33^ and starvation therapy.^34^ These studies demonstrate the great versatility of MnO_2_-based materials and their considerable potential as theranostics for cancer.

Recently, Mn nanomaterials have been used as redox responsive carriers of non-cytotoxic platinum-based prodrugs, Pt(iv) complexes, for cancer.^17,35,36^ Pt(iv) complexes act as chemotherapeutic prodrugs, since their cytotoxicity is hindered until reduced to Pt(ii) active drugs by reducing agents in diseased tissues.^11,37,38^ Cisplatin, a Pt(ii) chemotherapy drug, is clinically used in the treatment of several cancers, including lung and ovarian carcinomas, binding to DNA and inducing intra-strand cross-linking, which leads to apoptosis.^39^ The use of nanoparticles as Pt(iv) prodrug carriers has been shown to enhance the antitumor efficiency, while potentially decreasing the cytotoxicity towards healthy cells, when compared to the standard cisplatin therapy.^37^ The studies combining Mn-based nanomaterials with Pt(iv) prodrugs^11,17^ report very favourable outcomes, and show that both the MR and the therapeutic capabilities of the platforms are activated in the TME. However, preparation of these theranostic agents required multistep reactions, as well as introduction of other metals, such as iron or gold. As our group had previously prepared MnO_2_ nanostructures with optimal switchable MR properties using a fast and simple one-pot approach,^16^ we decided to employ a similar strategy to decrease the synthetic time and complexity of these materials.

**Fig. 1:**
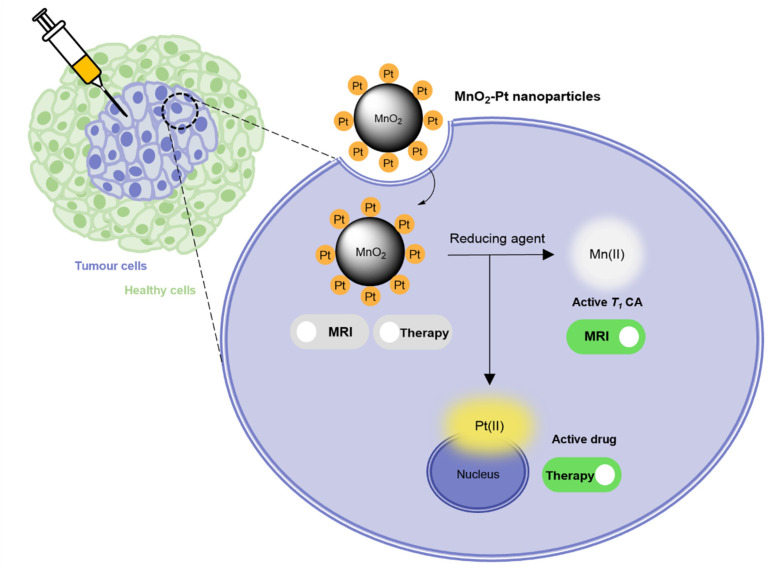
Schematic representation of the mechanism through which MnO_2_–Pt(iv) nanoparticles induce an MR switch and activation of apoptotic pathway in response to biologically available reducing agents in cancer cells.

In this work, a fast and facile one-pot ultrasonication reaction was employed to produce Pt(iv) prodrug-conjugated MnO_2_ nanoparticles as double redox responsive MRI nanotheranostics for cancer therapy. In the presence of reducing agents, the Pt(iv) complex is reduced, thus releasing the active chemotherapy drug cisplatin, which will induce cancer cell death. At the same time, the same reducing agents act on the MnO_2_ nanostructures to release free Mn(ii), hence inducing an off/ON *T*_1_ MR signal switch. As such, these smart theranostics are equipped with a redox double switch, as the redox environment triggers the off/ON switch of both the MR signal and chemotherapy efficacy (Fig. 1). These theranostics could potentially prove useful in the treatment of several types of cisplatin-resistant cancers, including lung and ovarian carcinomas, by circumventing inactivation resistance mechanisms usually dependent on GSH.^37^

## Results and discussion

### Synthesis and characterisation of MnO_2_–Pt(iv) nanoparticles

**Fig. 2:**
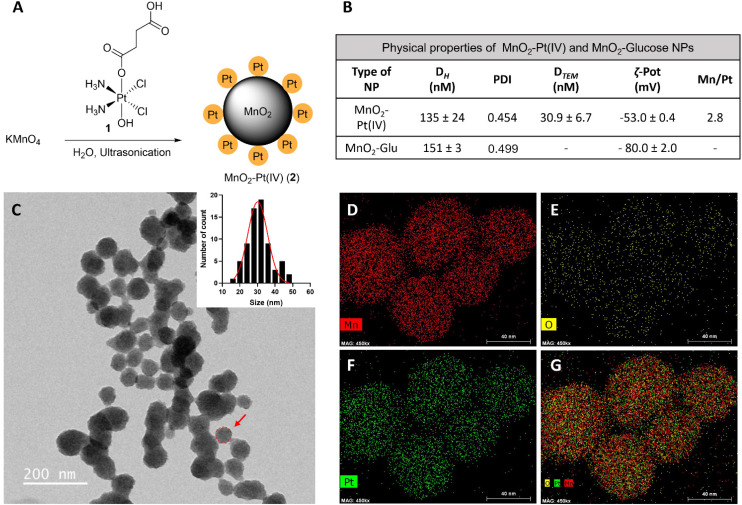
(A) Scheme of the preparation of MnO_2_–Pt(iv) nanostructures (2) by ultrasonication, from KMnO_4_ and Pt(iv) prodrug 1. (B) Table summarising the physical properties of MnO_2_–Pt(iv) and MnO_2_–Glucose nanoparticles.^16^ (C) TEM images of NPs and estimated nanoparticle size distribution. Scale bar represents 200 nm. Red circle and arrow represent the estimated diameter of the NPs considered for the size distribution. (D–G) STEM-EDX elemental distribution maps of the nanoparticles: (D) manganese, (E) oxygen, (F) platinum and (G) mix. Scale bar, 40 nm.

MnO_2_–Pt(iv) nanostructures were synthesised through a one-pot facile ultrasonication reaction from potassium permanganate (KMnO_4_) and a functionalised Pt(iv) prodrug complex^40^ (1, Fig. 2A), by adapting a previously published methodology,^16^ and purified by centrifugation. This is a complex reaction where Mn is reduced from (vii) to (iv), while the oxidation state of Pt is preserved as (iv). For this reaction to happen, the Pt precursor must be in excess as part of it is processed to reduce the Mn precursor. According to XPS data from the washings of the reaction and reaction controls (ESI, Fig. S4†), the succinic acid ligand and the Pt centre itself are involved in this reaction. The synthetic protocol was optimised in terms of the concentrations and ratio of Mn and Pt precursors, to obtain nanoparticles with optimal Mn : Pt ratio, to ensure both theranostic functions are viable and particles present an enhanced switchable MR performance (Table 1, ESI†). This optimisation study aimed to achieve relaxivity increases in the range of what has been reported before^16^ upon redox treatment. Additionally, the ratio of imaging agent to therapeutic effector (in this case Mn/Pt) is very important, as the acquisition of diagnostically relevant MR images requires a higher concentration of contrast agent than the concentration of chemotherapy drug necessary to induce cytotoxicity in pathological tissues. The dose of intravenous MnCl_2_ administered to mice for MRI can be as low as 6.6 mg kg^−1^ (52 μmol kg^−1^)^41,42^ and the LD_50_ is 38 mg kg^−1^ (302 μmol kg^−1^). A single dose of intravenous cisplatin administered to mice for chemotherapy is 5–6 mg kg^−1^ (17–20 μmol kg^−1^) with a LD_50_ of 6.6 mg kg^−1^ (22 μmol kg^−1^).^43^ As such, we aimed to synthesise nanoparticles with a Mn/Pt ratio of around 3.0. After optimisation of the synthesis of the MnO_2_–Pt(iv) nanoparticles, ICP results indicated that the theranostic platform had, on average, 2.8 Mn per every Pt (Fig. 2B), making them promising dual functional agents.

The hydrodynamic size (*D*_h_) and surface charge (ζ-pot) of these nanomaterials were measured using dynamic light scattering (DLS) (Fig. 2B and ESI Fig. S2†). The size of the MnO_2_–Pt(iv) nanostructures was 135 ± 24 nm and they exhibited negative surface charge in water at neutral pH (−53.0 ± 0.4 mV). These results are similar to those obtained for similar unfunctionalised MnO_2_ nanoparticles.^16^ Morphological analysis of these nanoparticles was carried out using transmission electron microscopy (TEM). The obtained images demonstrated that the synthesised nanoparticles presented a spherical structure (Fig. 2C). Scanning transmission electron microscopy with energy dispersive X-ray spectroscopy (STEM-EDXS) elemental maps (Fig. 2D–G) confirm that both Mn and Pt are present and well distributed in the nanostructures.

**Fig. 3:**
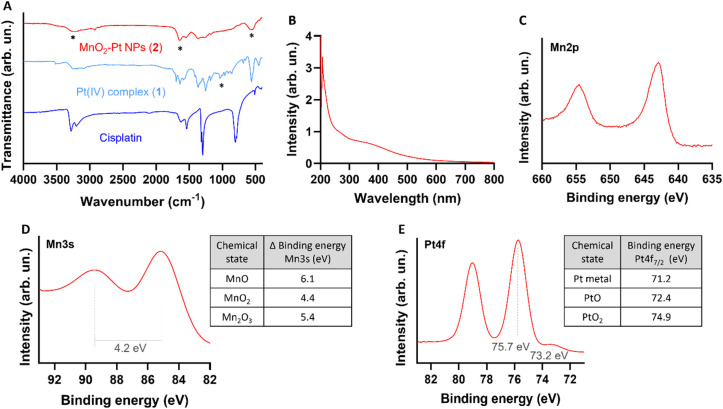
Physicochemical characterization of the nanostructures. (A) FTIR spectra of cisplatin (Pt(ii), active drug, dark blue), compound 1 (Pt(iv) precursor, light blue) and MnO_2_–Pt(iv) NPs (2, red). *Main peaks mentioned in the discussion. (B) UV-Vis absorption spectra of the nanostructures 2, showing a broad absorption around 350 nm. (C) Expanded region of XPS spectra of MnO_2_–Pt(iv) nanoparticles showing the peaks at region Mn2p.^44^ (D) Expanded Mn3s region of XPS spectra of MnO_2_–Pt(iv) NPs and table with reference magnitudes of peak splitting of different Mn oxidation states.^44^ (E) Expanded Pt4f region of XPS spectra and table with reference binding energies of common Pt states in the Pt4f region.^45^

Fourier transform infrared spectroscopy (FTIR) was then used to confirm the conjugation of Pt(iv) to the surface of the nanostructures. The obtained spectra for the MnO_2_–Pt(iv) nanoparticles shares similarities with the spectra of the Pt(iv) precursor, such as peaks at 1585 cm^−1^, corresponding to the bending of NH_3_ groups, and at 554 cm^−1^, attributed to the stretching of Pt–O bonds (Fig. 3A). However, the peak observed at 1040 cm^−1^ in the Pt(iv) precursor, corresponding to PtO–H bending, is not present in the FTIR spectra of the nanoparticles, indicating that the Pt(iv) complexes are bound to the nanoparticles through this functional group. A broad peak at 3000–3500 cm^−1^, from the bending and stretching vibration of C(=O)OH can also be observed. Ultraviolet-Visible (UV-Vis) spectroscopy shows a broad absorption in the UV region (∼350 nm, typical of MnO_2_ nanostructures) together with an exponential increase in absorbance as the wavelength decreases, consistent with subwavelength sized dielectric spheres (Fig. 3B).

The synthesis of the MnO_2_ nanostructures is a redox reaction in which Mn is reduced from +7 (from KMnO_4_) to +4 oxidation state. This reduction half-reaction requires an oxidation, that according to the precursors in the global reaction should come from the Pt(iv) precursor (either from Pt itself or its organic ligands). For these nanoparticles to display redox (and TME) responsive imaging and therapeutic properties, the Mn and the Pt in the NPs have to be in the +4 state, respectively. Thus, it is crucial to characterise in detail the redox state of Pt in the final nanostructures to guarantee that the Pt compound is still in the pro-drug (+4) state and has not been oxidized to the Pt(ii) drug or beyond. The redox state of the Mn in the nanoparticles also needs to be investigated, as the KMnO_4_ in the reaction could be reduced to Mn(iv), which would display the desired switchable imaging properties, but could also be reduced to a +2 state, in the form of MnO nanoparticles or Mn(ii) salts, which would mean that the final product would not present the desired responsive imaging properties. X-ray photoelectron spectroscopy (XPS) is a powerful technique to study the oxidation state of samples. The presence of Mn(iv) in the intact nanoparticles was confirmed by analysing the Mn2p and Mn3s peaks of the XPS spectra (ESI Fig. S3†). The multiplet-split components of the Mn2p region show two signals at 654.6 eV and 642.9 eV, with no observable peak or satellite feature typical of MnO being observed at ∼647 eV (Fig. 3C).^44^ The Mn3s peak shows two multiplet split components caused by the coupling of 3s electrons with 3d valence electrons (Fig. 3D). The magnitude of peak splitting for the tested nanoparticles is 4.2 eV, which is indicative of the presence of the Mn(iv) oxidation state (∼4.7 eV) instead of the Mn(ii) state (∼6 eV).^44^ The Pt4f region should show symmetric Pt4f peaks for both the Pt(ii) and Pt(iv) oxidation states, with the lowest binding energy peak appearing at ∼72.4 and ∼74.9, respectively.^45^ In the case of our nanostructures, the main peak of the Pt4f region, corresponding to 90% of the Pt in the sample, is observed at 75.7 eV, corresponding to the Pt(iv) oxidation state (Fig. 3E). The other small peak at 73.2 eV, corresponding to 10% of the measured Pt signal, is attributed to Pt(ii). Overall, the XPS results indicate that the synthesised MnO_2_–Pt(iv) nanoparticles are made of Mn(iv) and mostly constituted of Pt(iv) (>90%).

**Fig. 4:**
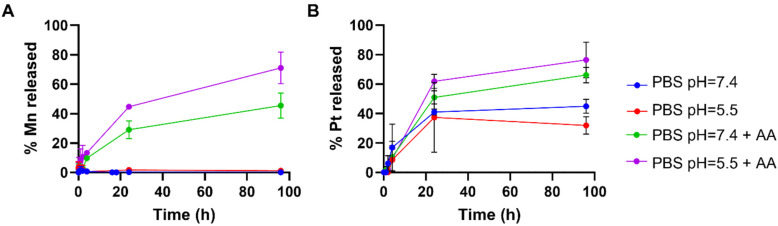
Time-dependent release of (A) Mn and (B) Pt from MnO_2_–Pt(iv) NPs (4 mM of Mn) in different PBS buffers with and without ascorbic acid (AA, 100 μM).

In order to study the responsiveness of the MnO_2_–Pt(iv) nanostructures to reducing agents, a drug release study was performed using a two-container setup under Fickean conditions (MWCO 6–8 kDa). While intact MnO_2_–Pt(iv) nanoparticles were too large to cross the membrane pores (>10 kDa), free Mn(ii) ions (released through the reduction of the nanostructures) and unbound Pt complexes (MW = 434.13 g mol^−1^) should easily cross the dialysis membranes. As such, the nanoparticles were placed inside the dialysis membrane and treated with PBS buffers at neutral or acidic pH, with or without ascorbic acid (100 μM). Aliquots of the outer solution were removed at different time points (2 min to 96 h), analysed by inductively coupled plasma atomic emission spectroscopy (ICP-OES) and compared to the amount of Mn/Pt present in the starting sample (Fig. 4A and B). Results showed no detectable release of Mn after 96 h of dialysis in conditions without reducing agent (shown in blue and red in Fig. 4A). In conditions containing reducing agents (ascorbic acid), an average of 42.6 and 71.4% of Mn was released from the nanoparticles at the end of the experiment in the buffers at pH = 7.4 and 5.5, respectively. The amount of Pt released from the nanoparticles was also measured, although distinction between released Pt(ii) and Pt(iv) was not possible using this method. While some Pt was detected in conditions without reducing agent, we assume this was due to desorption of Pt(iv) complexes from the surface of the nanostructures and not from reduction events, as no significant Pt(ii) was detected by XPS following storage in a PBS (pH = 7.4) suspension for over one month. Moreover, results show a similar trend where more Pt is released in the condition with pH = 5.5 and in the presence of a reducing agent, albeit in a faster manner. As the Pt(iv) prodrug should be present on the surface of the nanoparticles, reduction of the first “atomical” layer of the nanostructures should lead to the release of Pt, even when only minor Mn(ii) is released, thus explaining the faster release kinetics of Pt *vs.* Mn.

The stability of these nanoparticles in media was also evaluated by DLS, with results showing no significant aggregation of the nanoparticles in RPMI for up to 48 h (ESI Fig. S5†).

**Fig. 5:**
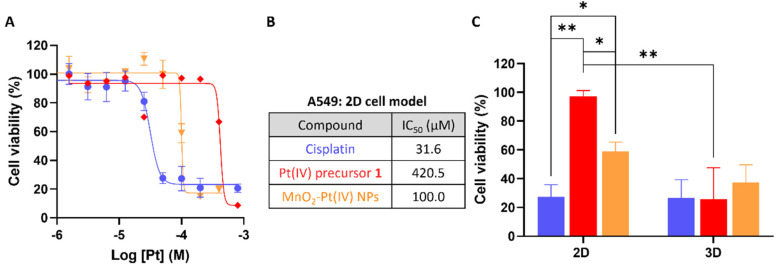
(A) Cell viability study employing 2D models of A549 cells after 48 h of treatment. (B) Calculated IC_50_ values (*n* = 3) for the different compounds. (C) Comparison of cell viability in 2D and 3D A549 cell cultures after 48 h of treatment with cisplatin, a Pt(iv) precursor, and NPs at a concentration of 100 μM of Pt (*n* = 3), *p < 0.04 and **p < 0.005.

To evaluate the effectiveness of these nanostructures as therapeutic effectors, *in vitro* toxicity studies were performed in A549 human non-small-cell lung carcinoma cells, which were chosen for this study since cisplatin has been used in the treatment of this type of cancer since the late 1970s.^46^ The cytotoxic effect of the MnO_2_–Pt(iv) was compared to that of the active drug cisplatin, the Pt(iv) precursor prodrug 1, and biocompatible MnO_2_–Glucose nanoparticles (Fig. 5B).^16^ Importantly, the MnO_2_–Glucose nanoparticles, as well as MnCl_2_ salts (ESI Fig. S6†), showed good biocompatibility in the concentration range tested.

The toxicity of the nanostructures (IC50 = 100.0 μM) was considerably higher than that of the precursor Pt(iv) prodrug (1, IC50 = 420.5 μM), indicating there is either a synergistic/cooperative effect between Mn and Pt, or that the delivery of prodrug 1 is more efficient in nanoparticle form (and thus higher intracellular concentrations of the prodrug are reached). To investigate this effect, A549 cells were co-incubated with prodrug 1 and a source of Mn (either MnCl_2_ or MnO_2_–Glucose nanoparticles). The results shown in Fig. S7 of the ESI† demonstrate that the presence of Mn enhances the cytotoxic effect of the prodrug. Similar tests performed with cisplatin showed no effect upon the addition of Mn, either in the salt or nanoparticle form. The cooperation between Pt prodrug 1 and Mn(ii) should be further investigated, but we hypothesize that the results shown here point towards an activation of Mn(ii)-mediated ferroptosis upon depletion of intracellular reducing agents (GSH and others). This possibility has already being proposed by other authors.^17,47^ The cell viability studies shown in Fig. 5A show a clear cytotoxicity difference between the active drug cisplatin *vs.* systems containing Pt(iv) prodrugs, indicating that these 2D cell models might not be reducing enough to completely convert the prodrugs into active drugs. As such, we performed cell viability studies in 3D cell models of the same A549 cell line since these can overcome some shortcomings of the 2D cancer cell cultures and better mimic the *in vivo* acidic and reductive tumour microenvironment.^48–51^ The results in Fig. 5C show that there was no significant difference in the cell viability following treatment with cisplatin, the Pt(iv) prodrug 1 and MnO_2_–Pt(iv) nanoparticles (100 μM of Pt) in 3D cell systems, demonstrating that these more complex 3D cell systems better recapitulate the reductive *in vivo* tumour microenvironment. The ability of the released Pt(ii) drug to interact with DNA was also explored. ICP-MS analysis of DNA samples extracted from cells incubated with the nanoparticles provided a reading of 12 ± 3 pg Pt μg^−1^ DNA, while no significant differences could be measured for Mn (Table S2†). These data once again confirm the reduction of the Pt prodrug into a Pt(ii) drug able to intercalate DNA. The 2D and 3D results together support the hypothesis that the use of prodrugs can help reduce off-target deleterious effects of chemotherapeutic drugs, without compromising on-target efficiency.

**Fig. 6:**
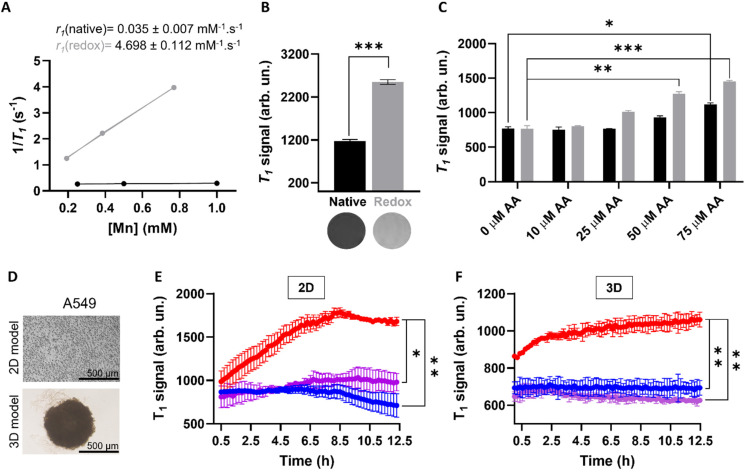
(A) Relaxometric studies before (black) and after (grey) addition of AA (10 mM) and corresponding measured relaxivity values, at 1.5 T. (B) *T*_1_-Weighted MRI phantoms of NPs dispersions (bottom) and corresponding signal before (black) and after (grey) redox treatment, [Mn] = 700 μM, [AA] = 10 mM, at 3.0 T, ***p < 0.0001. (C) MR signals of NP dispersions in the presence of different concentrations of AA (0–75 μM) at pH = 7.4 (black) and 5.5 (grey), at 3.0 T, *p = 0.01, **p = 0.007 and ***p < 0.0001. (D) Representative images of 2D (top) and 3D (bottom) cell cultures used for *in vitro* imaging and cell viability studies. Scale bar represents 500 μm. (E) and (F), MR *T*_1_ signal evolution of (E) 2D and (F) 3D A549 cells treated with MnO_2_–Pt NPs (637 μM and 500 μM of Mn, respectively) over time, at 3.0 T. Blue: cells; purple: NPs in media, red: cells treated with NPs, *p = 0.013, **p < 0.005.

In order to investigate whether the release of Mn(ii) from the MnO_2_–Pt(iv) nanostructures would potentiate an MR off/ON switch, relaxometry studies at 1.5 T were carried out in the presence and absence of ascorbic acid as a reducing agent (Fig. 6A). The relaxometric properties (particularly the longitudinal relaxivity) of the synthesised probe increased considerably (136-fold) following addition of ascorbic acid (10 mM, native *r*_1_ = 0.035 *vs.* redox *r*_1_ = 4.698 mM^−1^ s^−1^), an increase similar to that previously reported for this type of material.^16^ To confirm these results, *T*_1_-weighted MR phantom images of MnO_2_–Pt(iv) nanostructures (500 μM of Mn) were acquired using an MR scanner at the clinical field of 3.0 T, before and after the addition of ascorbic acid (10 mM). The obtained MR images clearly show an off/ON behaviour for these nanostructures after treatment with excess reducing agent (Fig. 6B). To evaluate whether the nanoparticles would still display redox responsive MR switchable properties after addition of biologically relevant concentrations of reducing agents (micromolar range), a more in-depth MR study was designed. Firstly, MnO_2_–Pt(iv) nanostructures (500 μM of Mn) were treated with a wide range of ascorbic acid concentrations (0.1 μM to 10 mM) at pH = 7.4 and imaged at 3.0 T. From the obtained MR phantoms (ESI, Fig. S8†), it becomes clear that the imaging switch occurs at ascorbic acid concentrations between 10 and 100 μM, at this pH. A smaller range 10–75 μM was then further explored at two different pH values (7.4 and 5.5) through *T*_1_-weighted MR images. The results showed that the minimum concentrations of ascorbic acid required to induce a statistically relevant off/ON imaging switch were 50 and 75 μM, at pH = 5.5 and 7.4, respectively (Fig. 6C). As such, in accordance with the drug release study, the redox responsive imaging feature of the nanostructures not only depends on the concentration of reducing agent, but also on the pH. Having a redox-responsive MR switch that is enhanced at lower pH is very beneficial in this case, as these MnO_2_–Pt(iv) NPs aim to target the tumour microenvironment, which is both acidic and reductive. Moreover, the required concentration of reducing agent is within the biologically relevant range,^18^ particularly taking into account that intracellular reducing agents other than ascorbic acid are also capable of reducing these MnO_2_–Pt(iv) nanoparticles (ESI, Fig. S9†). The relaxometric properties of these nanomaterials were also evaluated in murine serum over time at 9.4 T (ESI, Fig. S10†). Results showed no significant variation of the relaxivity of MnO_2_–Pt(iv) NPs for up to 24 h, indicating that the nanostructures are stable in serum, though they were found to bind BSA (ESI, Fig. S11†).

Going a step further, these nanostrutures were used as redox responsive MR contrast agents *in vitro* in 2D and 3D models of lung carcinoma cell line A549 (Fig. 6D). First, the 2D cell model was investigated by plating A549 cells in a custom 96-well plate. The cells were incubated for 24 h and then treated with MnO_2_–Pt(iv) nanoparticles and immediately imaged. *T*_1_-weighted phantom images were acquired every 12 min over 12 h (ESI, Fig. S12†). Results show that the *T*_1_ signal increased significantly (240%) in the cells treated with MnO_2_–Pt(iv) nanoparticles when compared to cells alone and nanoparticles in media (Fig. 6E). This infers that the nanoparticles were reduced over time by the A549 cells, leading to the release of Mn(ii) ions and consequent MR enhancement that plateaus after around 8.5 h. The MR signal evolution following treatment with MnO_2_–Pt(iv) nanoparticles was also studied in A549 spheroids (3D model), as these better mimic the reductive and hypoxic conditions characteristic of most solid tumour microenvironments. The spheroids were grown for 3 days, then carefully transferred into a custom MR plate, treated with MnO_2_–Pt(iv) nanostructures and immediately introduced in the MR scanner and imaged every 12 min over 12 h (Fig. 6F). These results show a similar increase in the *T*_1_ signal over time shown by 2D cultures treated with the responsive nanoparticles, demonstrating that cells grown in 3D conditions are still able to interact and process the nanostructures.

**Fig. 7:**
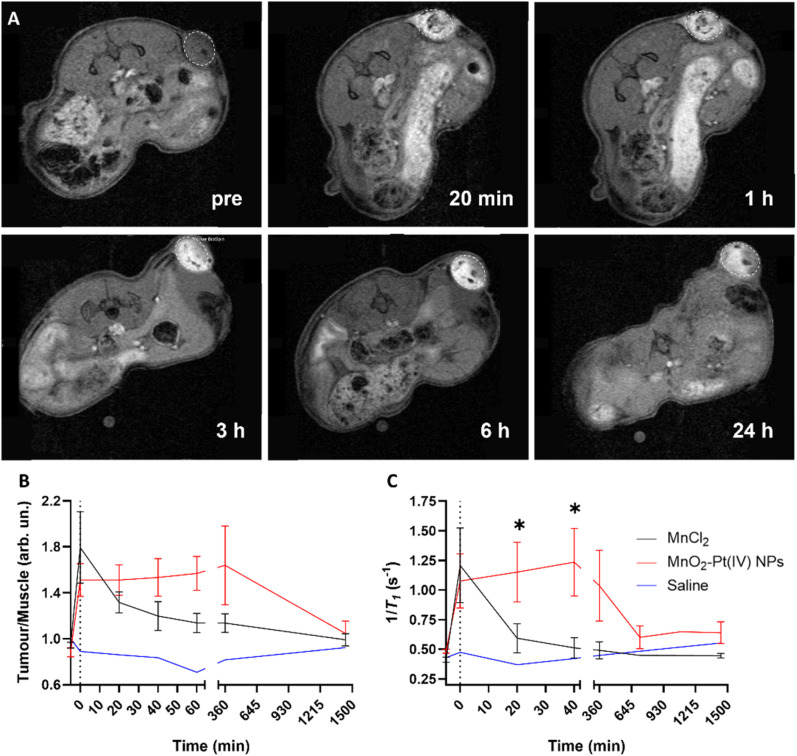
(A) *T*_1_-Weighted axial MR images of tumour-bearing Balb/c nude mice before (pre) and 20 min, 1 h, 3 h, 6 h and 24 h after intratumoral injection of MnO_2_–Pt(iv) NPs, using a 9.4 T MR scanner. White circles highlight tumour sites. (B) Corresponding *T*_1_ signal tumour/muscle ratio obtained using a FLASH sequence and (C) Local *T*_1_ values of tumour and muscle tissues of mice treated with MnO_2_–Pt(iv) NPs (*n* = 5), MnCl_2_ (*n* = 6) and saline (*n* = 1), obtained using a *T*_1_ mapping sequence on the 9.4 T MR scanner, showing mean and errors (SEM), *p < 0.045.

These results indicate that the MnO_2_–Pt(iv) nanostructures work as responsive MR agents *in vitro*. Subsequently, *in vivo* studies were performed on A549 tumour-bearing Balb/c nude mice, using a 9.4 T MR scanner. MnO_2_–Pt(iv) nanostructures, or MnCl_2_ (6 mg kg^−1^ of Mn) as control, were injected directly into the tumour tissue and axial *T*_1_-weighted images were acquired pre-injection and at appropriate intervals (0 min, 20 min, 40 min, 60 min, 3 h, 6 h and 24 h) post-injection (Fig. 7 and ESI Fig. S13†). A significant tumour signal enhancement was observed immediately after the injection of MnO_2_–Pt(iv) nanoparticles, suggesting that the nanoparticles were readily reduced in the tumour. Importantly, the *T*_1_ signal enhancement in the tumour could still be detected 24 h post-injection of the nanoparticles, but not of MnCl_2_, which was cleared much faster (3 h) from the tumour tissue (ESI Fig. 13†). Comparison of the corresponding *T*_1_ signal intensity at the tumour site *vs.* muscle, confirmed that the MnO_2_–Pt(iv) nanostructures induced a brighter and longer-lasting tumour MR enhancement than conventional MnCl_2_ (Fig. 7B). *T*_1_ mapping studies were also performed at different time points (Fig. 7C and ESI Fig. S14†). Since a shorter *T*_1_ (longer 1/*T*_1_) is associated with a higher MR signal enhancement, it is expected that samples with brighter tumour contrast (*e.g.*, tumours injected with MnO_2_–Pt(iv) NPs) would present shorter *T*_1_ values. As expected, the obtained *T*_1_ values of tumours injected with MnO_2_–Pt(iv) nanoparticles were significantly shorter than those of tumours injected with MnCl_2_ and/or saline. A considerable *T*_1_ value decrease in nanoparticle-treated tumours was observed immediately following injection (average per group of 1181.9 ms *vs.* 2081.74 ms, 43% decrease *vs.* pre-injection) and was maintained for at least 6 h post-injection (1243.6 ms *vs.* 2081.74 ms, 40% decrease compared to pre-injection). On the other hand, the tumour *T*_1_ following treatment with MnCl_2_ was lowest shortly after injection (1279.2 ms, 48% decrease at 0 min post-injection) and quickly recovered with time (2156.0 ms, 12% decrease at 40 min post-injection).

These results confirmed that MnO_2_–Pt(iv) nanostructures are effective *T*_1_ contrast agents *in vivo* and have great potential for clinical use.

## Conclusion

In this work we synthesised redox responsive MnO_2_–Pt(iv) MR nanotheranostics for cancer therapy, using a fast and simple one-pot ultrasonication methodology. After thorough characterisation, *in vitro* studies confirmed that the nanostructures can induce apoptosis in cancer cell lines, presenting an IC_50_ of 100.0 μM in 2D cell systems of A549 cell line. Moreover, we proved that the MnO_2_–Pt(iv) nanostructures are at least as efficient at inducing cell death as cisplatin in more complex and more reductive 3D cell models.

These nanoparticles exhibit a strong off/ON MR behaviour in response to reducing agents, with longitudinal relaxivity (*r*_1_) values increasing 136-fold upon treatment with ascorbic acid. This off/ON MR behaviour was also observed *in vitro*, with the *T*_1_ signal of A549 2D and 3D cell models being enhanced over time upon addition of the MnO_2_–Pt(iv) nanoparticles. Importantly, these nanostructures led to a strong and long-lasting *T*_1_ signal enhancement *in vivo*, with a 240% *T*_1_ enhancement observed at 3 h post-injection (*vs.* 110% for MnCl_2_) following intra-tumoural injection. While systemic imaging and therapeutic studies still need to be performed, these results indicate that MnO_2_–Pt(iv) nanostructures present great potential as redox responsive dual switch MR theranostics for cancer therapy.

## Materials and methods

### General

Cisplatin was purchased from TCI Chemicals (Zwijndrecht, Belgium). Dulbecco's Modified Eagle's medium/Nutrient Mixture F-12 (DMEM-F12) was purchased from Gibcol Solutions Ltd (Auckland, New Zealand). Penicillin–Streptomycin solution was purchased from Biotecnomica Unipessoal Lda (São Mamede Infesta, Portugal). Fetal bovine serum, trypsin and was purchased from ThermoFisher (Massachusetts, USA). BODIPY581/591-C11 was purchased from LabClinics S. A. (Barcelona, Spain). Other chemicals and reagents were purchased from Sigma-Aldrich (Missouri, USA). All purchased products were used as supplied without any further purification. The water used in all experiments was ultra-pure MilliQ water (18 MΩ).

Hydrodynamic size and surface charge studies were performed on a Horiba nanoPartica SZ-100 instrument. A JEOL 2010 transmission electron microscope (JEM-2100-HT) working at 200 keV was used to image the nanoparticles. The TEM samples were prepared by depositing nanoparticle aqueous solutions (7 mL) onto 400 mesh carbon coated copper TEM grids (EM Resolutions Ltd, UK) and dried at room temperature for 24 h before use. UV/Vis spectra were recorded using a Shimadzu UV-2550 UV/Vis spectrophotometer. FTIR spectra were recorded using a VERTEX 80v vacuum FTIR spectrometer. A spectrometer ICPE-9000 was used to measure the concentration of Mn and Pt. XPS measurements were performed on an ESCALAB™ QXi X-ray Photoelectron Spectrometer. The XPS samples were prepared by drop casting onto clean silicon wafers.

### Synthesis of Pt(iv) precursor

Pt(iv) prodrug oxoplatin (*cis, cis, trans*-diamminedichlorodihydroxyplatinum(iv), DHC) was prepared according to previously published methodologies. Briefly, hydrogen peroxide (H_2_O_2_, 30% in water, 70 equiv.) was added dropwise to a bright yellow suspension of cisplatin (1.80 mmol, 1 equiv.) in water inside a microwave vessel. The reaction mixture was heated to 70 °C for 15 min in a CEM Discover SP microwave. The reaction mixture was cooled down and then the solvent was removed *in vacuo*. The residue was sequentially suspended in ethanol and diethyl ether to afford a light-yellow powder. Recrystallisation from water provided oxoplatin as bright yellow crystals (1.11 mmol, 61%). Product was characterised by XRD (ESI, Fig. S15†), FTIR (cm^−1^): 3520 (*ν*_O–H_), 1040 (*δ*_PtO–H_) and 552 (*ν*_PtO_), and EA: calculated (%) for Cl_2_H_8_O_2_N_2_Pt: C 0.00, H 2.41, N 8.39; found (%): C 0.00, H 2.44, N 8.52.

The modified Pt(iv) complex (1) was prepared by adding succinic anhydride (0.20 mmol, 1.1 equiv.) to a suspension of oxoplatin (0.18 mmol, 1 equiv.) in dimethylsulfoxide (DMSO).^52^ The mixture was stirred overnight at room temperature. The solvent was evaporated, and the resulting solid was washed with ice cold acetone, to yield complex 1 as a pale-yellow solid (0.10 mmol, 54%). Product was characterised by FTIR (cm^−1^): 3500–3000 (broad, *ν*_C(=O)OH_), 1585 (*δ*_NH3_), 1040 (*δ*_PtO–H_) and 554 (*ν*_PtO_).

### Synthesis of MnO_2_–Pt(iv) nanoparticles

Pt(iv) precursor complex 1 (0.046 mmol, 3.5 equiv.) was dissolved in MilliQ water (8 mL). KMnO_4_ (0.013 mmol, 1.0 equiv.) was added, and the resulting solution was immediately sonicated for 2 min at 25% power, in a Branson 250 Digital sonifier equipped with a 1/8′′ microtip providing an amplitude range of 116–494. The solutions were then cooled down and centrifuged at 10 000 rpm for 15 min. The supernatant was discarded, and the pellet re-suspended in 1 mL of water. This process was repeated twice more and finally the pellet was resuspended in 1 mL of water. This solution was then centrifuged for 90 s at 3000 rpm to remove large aggregates. The supernatant was kept and stored until further use.

### Release studies

Drug release studies were performed using Pur-A-Lyzer™ Mini Dialysis kit (MWCO 6–8 kDa). MnO_2_–Pt(iv) nanoparticles (100 μL, 4.1 mM of Mn) were incubated inside the dialysis membrane eppendorfs with 100 μL of different buffers: PBS at pH = 7.4, PBS at pH = 5.5, PBS at pH = 7.4 with ascorbic acid (100 μM) and PBS at pH = 5.5 with ascorbic acid (100 μM). The membrane eppendorfs were closed and placed inside falcon tubes filled with the corresponding buffer (10 mL). At different time points (2 min to 96 h), 1 mL of the outside solution was removed and analysed by ICP. The volume was then adjusted by adding 1 mL of the appropriate buffer to the outside solution. This dilution was taken into account when calculating the % Mn and Pt released from the nanoparticles.

### Relaxivity measurements

*T*_1_ relaxation times were measured with a Minispec mq60 relaxometer working at 1.5 T. At least three concentrations were measured for each sample and all experiments were performed at 37 °C and pH = 7.4. Standard inversion recovery protocols were used to measure the longitudinal relaxation time (*T*_1_). The longitudinal relaxivity value (*r*_1_, in mM^−1^ s^−1^) was calculated as the slope of the curve fitting 1/*T*_1_ (in s^−1^) *vs.* Mn concentration (in mM).

### *In vitro* cellular cytotoxicity assays

For 2D cell viability studies, A549 cells were seeded in 96-well plates at a density of 5000 cells per well in 100 μL of complete DMEM F-12 medium and incubated in a 5% CO_2_ atmosphere at 37 °C for 24 h. The culture medium was then removed and replaced with 100 μL of medium containing compounds of interest at different concentrations. The cells were incubated for 48 h. Resazurine was added to each well and the cells were incubated for another 4 h to allow viable cells to reduce the non-fluorescent blue resazurin to red fluorescent dye resorufin. The fluorescence was measured at 590 nm by using a Biotek Synergy H1 Microtiter Plate Reader (*λ*_ex_ = 560 nm, *λ*_em_ = 590 nm).

For 3D cell viability studies, A549 cells were seeded in BIOFLOAT™ 96-well plates (purchased from faCellitate) at 10 000 cells per well in 200 μL of complete DMEM F-12 medium. The plates were centrifuged at 1200 rpm for 5 min and then incubated for 3 days; medium was changed as needed. The spheroids were photographed using phase-contrast microscope. The culture medium was then replaced with 200 μL of medium containing compounds of interest at different concentrations. The cells were incubated for 48 h and were then photographed. Resazurine was added to each well and the cells were incubated for another 12 h. The fluorescence was measured at 590 nm by using a Biotek Synergy H1 Microtiter Plate Reader (*λ*_ex_ = 560 nm, *λ*_em_ = 590 nm).

### *In vitro* Pt-DNA adduct formation

For 2D cell culture: A549 cells were seeded in 6-well plates at a density of 50 000 cells per well in 1 mL of complete DMEM F-12 medium and incubated in a 5% CO_2_ atmosphere at 37 °C for 24 h. The culture medium was then removed and replaced with 500 μL of medium containing and MnO_2_–Pt(iv) nanoparticles at a Pt concentration of 0.167 mM. The cells were incubated for 8 h. Following incubation, cells were washed 3× with PBS. Cells were then detached using a standard trypsinization method and the DNA extracted using a PureLink™ Genomic DNA Mini Kit (Invitrogen). DNA concentration was determined using a NanoDrop 2000 spectrophotometer.

For 3D cell culture: A549 cells were seeded in spheroid appropriate 96-well plates at density of 10 000 cells per well in 200 μL of complete DMEM F-12 medium and centrifuged for 2 minutes at 1500 RPM. Plates were then incubated in a 5% CO_2_ atmosphere at 37 °C for 72 h. 100 μL was carefully removed and replaced with MnO_2_–Pt(iv) nanoparticles to a final Pt concentration of 0.167 mM. The cells were incubated for 8 h. Following incubation, spheroids were washed 3× with PBS. DNA was then extracted using a PureLink™ Genomic DNA Mini Kit (Invitrogen). DNA concentration was determined using a NanoDrop 2000 spectrophotometer.

### *In vivo* studies

All animal experiments were performed in the U.K. in accordance with the United Kingdom Home Office Animal (scientific procedures) Act 1986, and in accordance with the Guidelines for Care and Use of Laboratory Animals of King's College London. All experiments were approved by the Animal Ethics Committee of “KCL – Waterloo Campus”.

A549 (5 × 10^6^) human lung cancer cells in Dulbecco's PBS (100 μl) were injected subcutaneously into the flank of female Balb/c nu/nu mice aged 6–9 weeks (Charles rivers Laboratories). Tumour growth was monitored daily using an electronic calliper and the volume was calculated using the equation below, where*h*, *w*, and *l* represent, height, width, and length, respectively.
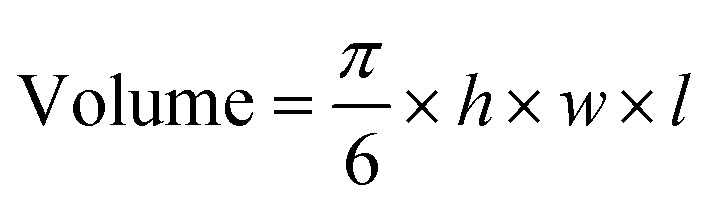


### Magnetic resonance imaging

Phantom imaging: MR imaging was performed in a 3.0 T horizontal bore MR Solutions Benchtop MRI system (Guildford, UK) equipped with 48 G cm^−1^ actively shielded gradients. For imaging the sample, a 56 mm diameter quadrature birdcage coil was used in transmit/receive mode. All MR images of the phantoms were acquired with an image matrix 256 × 252, FOV 60 × 60 mm, 3 slices with a slice thickness of 1 mm and with no slice gap. For *T*_1_-weighted imaging, a fast spin echo based (FSE) sequence with the following parameters was used: echo time (TE) = 11 ms, repetition time (TR) = 720 ms, number of averages (NA) = 32.

For functional studies, a customised 3D printed PLA holder containing 16 wells surrounded by a compartment filled with water was employed. Samples (500 μM of Mn in PBS pH = 7.4, 200 μL) were placed inside the wells, with or without reducing agents (ascorbic acid, glutathione or hydrogen peroxide, 0.001–10 mM).

For *in vitro* studies employing 2D cell models, a 96-well plate was custom cut to fit inside the MR coil, and 50 000 cells per well were seeded and incubated at 37 °C overnight. After 24 h, the cells were imaged (*t* = 0 min). The cells were then treated with the MnO_2_–Pt(iv) nanoparticles (600 μM of Mn) and immediately imaged using *T*_1_-weighted scans every 12 minutes over 12 h. A fast spin echo based (FSE) sequence with the following parameters was used: TE = 11 ms, TR = 720 ms, NA = 32.

For *in vitro* studies employing 3D cell models, a 1% agarose solution (%m/v, in H_2_O) was added to each well of a customised 3D printed holder. The holder was placed in the fridge for 30 min for the agarose to set. Then, one A549 spheroid (3 days of growth) was placed inside each well and imaged (*t* = 0 min). The spheroids were then treated with the MnO_2_–Pt(iv) nanoparticles (500 μM of Mn) and immediately imaged using *T*_1_-weighted every 12 minutes over 12 h. A fast spin echo based (FSE) sequence with the following parameters was used: TE = 11 ms, TR = 720 ms, NA = 32.

The *in vivo* MR studies were carried out when the volume of mice tumours reached ∼50 mm.^3^ Mice were placed under isoflurane anaesthesia (1.5–2% in O_2_) and kept at 37 °C throughout the measurements and were intratumorally injected with a dose of 6 mg kg^−1^ of Mn of either MnO_2_–Pt(iv) nanoparticles, MnCl_2_, or saline. *T*_1_-Weighted images were acquired using a Bruker Avance III 400 MHz (9.4 T) (Bruker, Germany) before the intratumoral injection and 1, 6 and 24 h post injection (p.i.). A dynamic acquisition was carried out during the first hour p.i. *T*_1_-weighted images were acquired with the axial and coronal orientation using a FLASH sequence. The following acquisition parameters were used in all *T*_1_-weighted image acquisitions: TR = 600 ms, TE = 1.6 ms, field of view (FOV) = 25 mm × 25 mm, matrix size = 256 × 256, slice thickness = 1 mm (5 slices, gap = 0). *T*_1_ mapping images were acquired in the same conditions before the intratumoral injection and 20 and 40 min and 6 and 24 h post injection using a RARE sequence. The following acquisition parameters were used: repetition time (TR) = 0.3–5.5 s (6 measurements), echo time (TE) = 6.5 ms, field of view (FOV) = 25 mm × 25 mm, matrix size = 128 × 128, slice thickness = 1 mm (5 slices, gap = 0). Data were analysed using TopSpin software version 2.1 (Bruker, Germany).

## Abbreviations

2D: Two dimensional
3D: Three dimensional
BSA: Bovine serum albumin
*D*_H_
: Hydrodynamic size
DLS: Dynamic light scattering
FTIR: Fourier transform infrared spectroscopy
ICP-OES: Inductively coupled plasma atomic emission spectroscopy
MRI: Magnetic resonance imaging
MW: Molecular weight
MWCO: Molecular weight cut-off
NPs: Nanoparticles
SEM: Standard error of the mean
TEM: Transmission electron microscopy
TME: Tumour microenvironment
UV-Vis: Ultraviolet visible spectroscopy
XPS: X-ray photoelectron spectroscopy.

## Author contributions

JG, GJS and MB, conceptualization, supervision, project administration, funding acquisition. BB, MRR, AJW, OT and VR, investigation. BB, MSC, NG, TWP, TRE, MB, GJS and JG, methodology. BB, TRE, MB, GJS and JG, visualization, formal analysis. BB, MB, GJS and JG, writing – original draft. All authors, writing – review and editing.

## Conflicts of interest

The authors declare no conflicts of interest.

## Supplementary Material

NR-015-D3NR00076A-s001
