# Supplementary material for: Redox double-switch cancer theranostics through Pt(iv) functionalised manganese dioxide nanostructures
Source: Nanoscale. 2023 Jun 13;15(25):10763–75. doi: 10.1039/d3nr00076a (PMC10311465; doi:10.1039/d3nr00076a)
Supplement: NR-015-D3NR00076A-s001 [file NR-015-D3NR00076A-s001.pdf]

Electronic Supplementary Material (ESI) for Nanoscale.
This journal is © The Royal Society of Chemistry 2023

Title: Redox double switch theranostics against cancer: Pt(IV) prodrug-functionalised MnO_2 nanostructures

Authors: Beatriz Brito,[a,b,c] Maria Rosaria Ruggiero,[a] Thomas W. Price,[a] Milene da Costa Silva,[c] Núria Genicio,[c] Annah J. Wilson,[a] Olga Tyurina,[a] Veronika Rosecker,[a] Thomas R. Eykyn,[a] Manuel Bañobre-López[c], Graeme J. Stasiuk[a] and Juan Gallo[c]

Affiliations: [a]Department of Imaging Chemistry and Biology, School of Biomedical Engineering and Imaging Sciences, King's College London, Strand, WC2R 2LS London, UK graeme.stasiuk@kcl.ac.uk

[b]School of Life Sciences, Faculty of Health Sciences, University of Hull, Cottingham Road, HU6 7RX Hull, UK

[c]Advanced Magnetic Theranostic Nanostructures Lab, International Iberian Nanotechnology Laboratory, Av. Mestre José Veiga, 4715-330 Braga, juan.gallo@inl.int , manuel.banobre@inl.int

Supporting Information

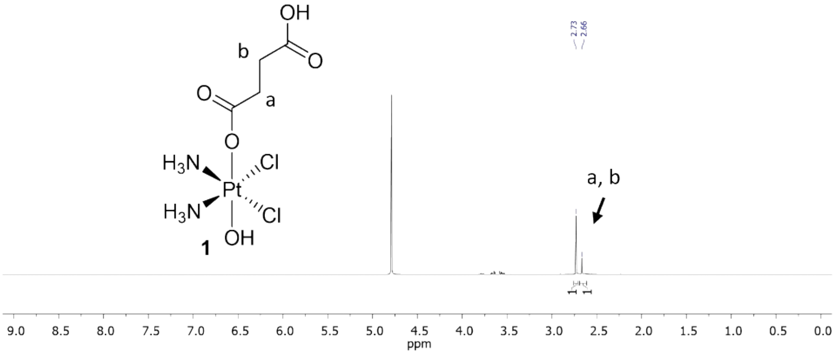

Figure S1. ^{1}H NMR of Pt(IV) complex **1**, in D_2O, at 400 MHz.

Table S1. Optimisation of the preparation of MnO_2-Pt NPs. Reaction conditions, hydrodynamic size (D_H), surface charge (ζ-Pot), and ICP measurements. Relaxivity measurements performed in the ajbsence (native) and presence of ascorbic acid (10 mM, 1.5 T).

Reaction	$[KMnO_4]$ (mM)	Mn/Pt reaction	D_H (nm)	ζ-Pot (mV)	Mn/Pt ICP	Native r_1 ($mM^{-1}.s^{-1}$)	Redox r_1 ($mM^{-1}.s^{-1}$)	Fold increase
A	9	0.50	187 ± 54	-43.0 ± 1.5	0.5	0.046 ± 0.002	0.343 ± 0.026	7
B	1.6	2	198 ± 44	-46.3 ± 1.4	9.1	0.066 ± 0.007	7.201 ± 0.351	108
C		1	123 ± 38	-47.8 ± 1.3	8.3	0.078 ± 0.006	6.147 ± 0.141	79
D		0.70	160 ± 61	-52.4 ± 0.9	4.0	0.057 ± 0.001	5.666 ± 0.134	100
E		0.5	156 ± 37	-45.9 ± 0.9	4.0	0.104 ± 0.005	6.236 ± 0.227	60
F		0.25	75 ± 21	-53.2 ± 2.1	5.3	0.035 ± 0.007	4.698 ± 0.112	136
G			145 ± 35	-54.6 ± 1.9	4.5	0.070 ± 0.002	5.747 ± 0.077	82
H			135 ± 35	-26.2 ± 0.3	4.5	0.067 ± 0.011	7.258 ± 0.158	108
I			140 ± 20	-53.0 ± 0.4	2.8	0.065 ± 0.003	4.700 ± 0.008	72

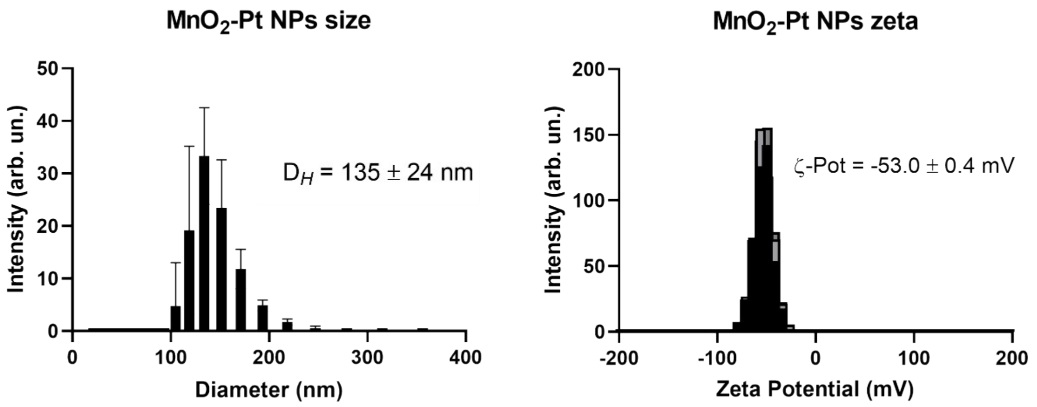

Figure S2. DLS and zeta measurements of MnO_2-Pt(IV) NPs.

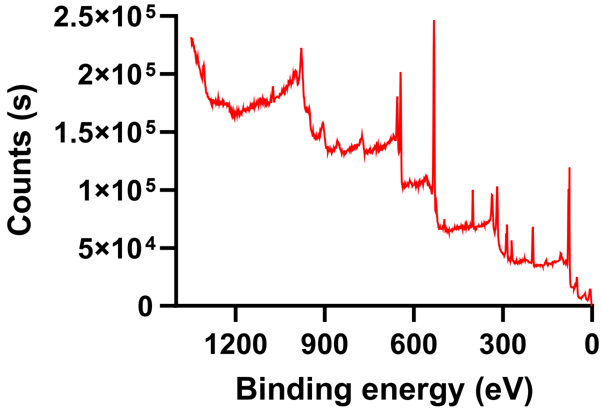

Figure S3. XPS spectra of MnO_2-Pt(IV) NPs.

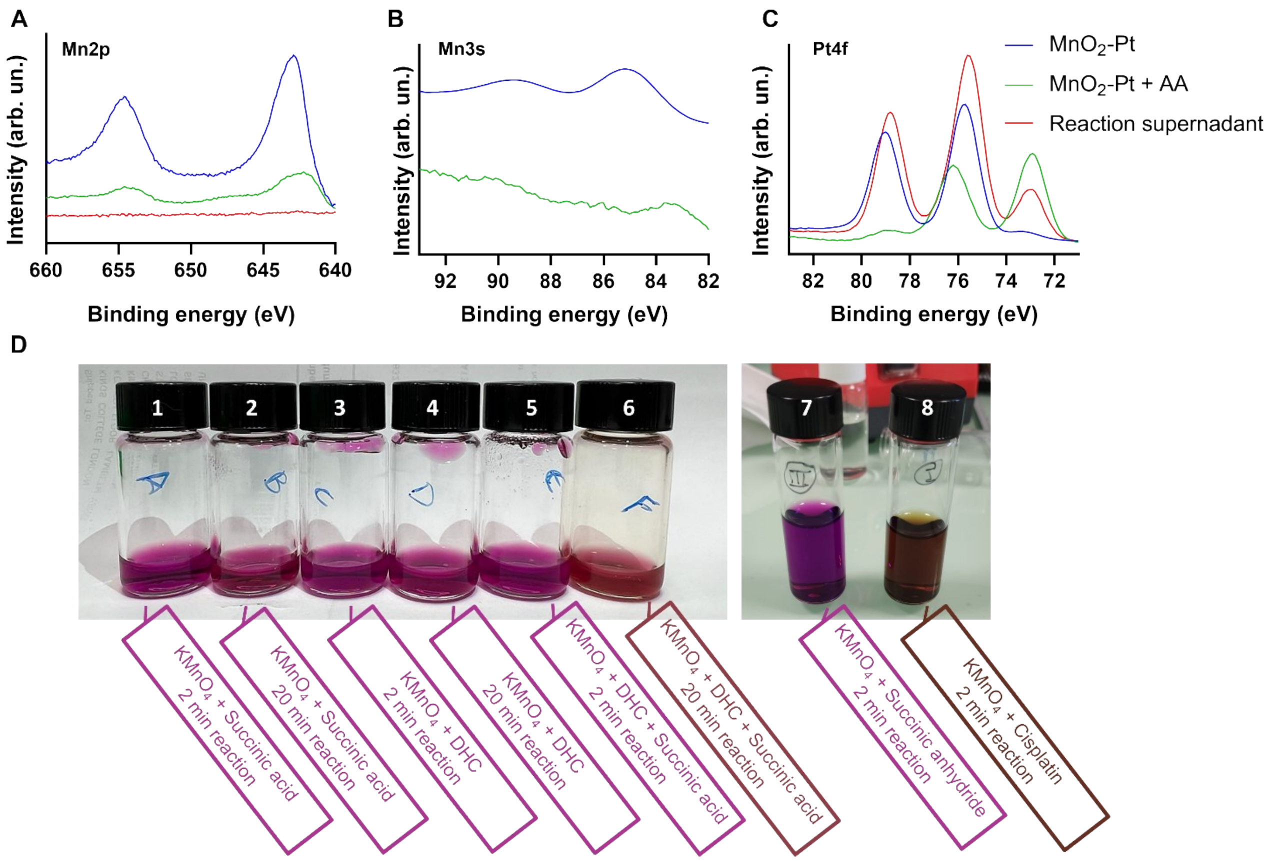

Figure S4. Expanded **A)** Mn2p, **B)** Mn3s and **C)** Pt4f regions of XPS spectra of MnO_2-Pt(IV) nanoparticles. Blue line: MnO_2-Pt(IV) NPs (pH = 7.4); green line: MnO_2-Pt(IV) NPs (pH = 7.4, 100 μM AA); red line: reaction supernatant obtained during purification of MnO_2-Pt(IV) NPs (pH = 7.4). D) Control $KMnO_4$ ultrasonication reactions with different ligands. Reaction mixtures that remained purple (1-5 and 7) show that Mn(VII) was not reduced, and nanoparticles were not formed. Reaction mixtures that turned brown after ultrasonication (6 and 8) indicate that the Mn(VII) in solution was reduced into nanoparticles.

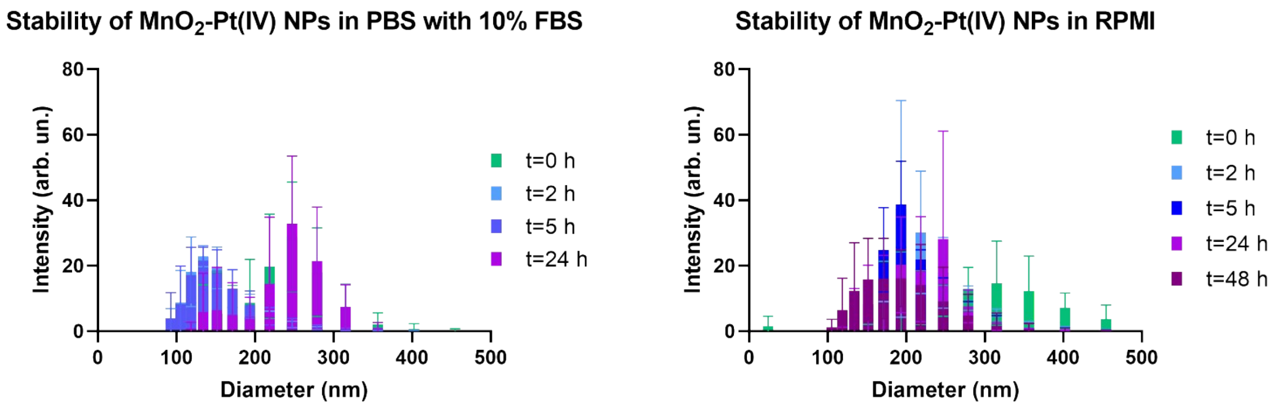

Figure S5. DLS measurements of MnO_2-Pt nanoparticles in PBS with 10% FBS (left) or RPMI with 10% FBS (right) at different time points.

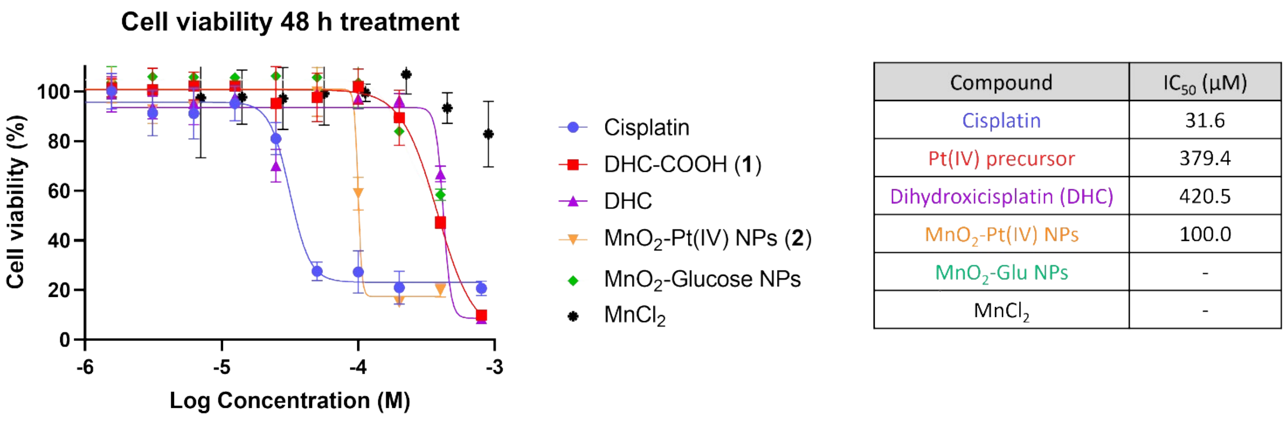

Figure S6. Cell viability study employing 2D model of A549 cells after 48 h of treatments.

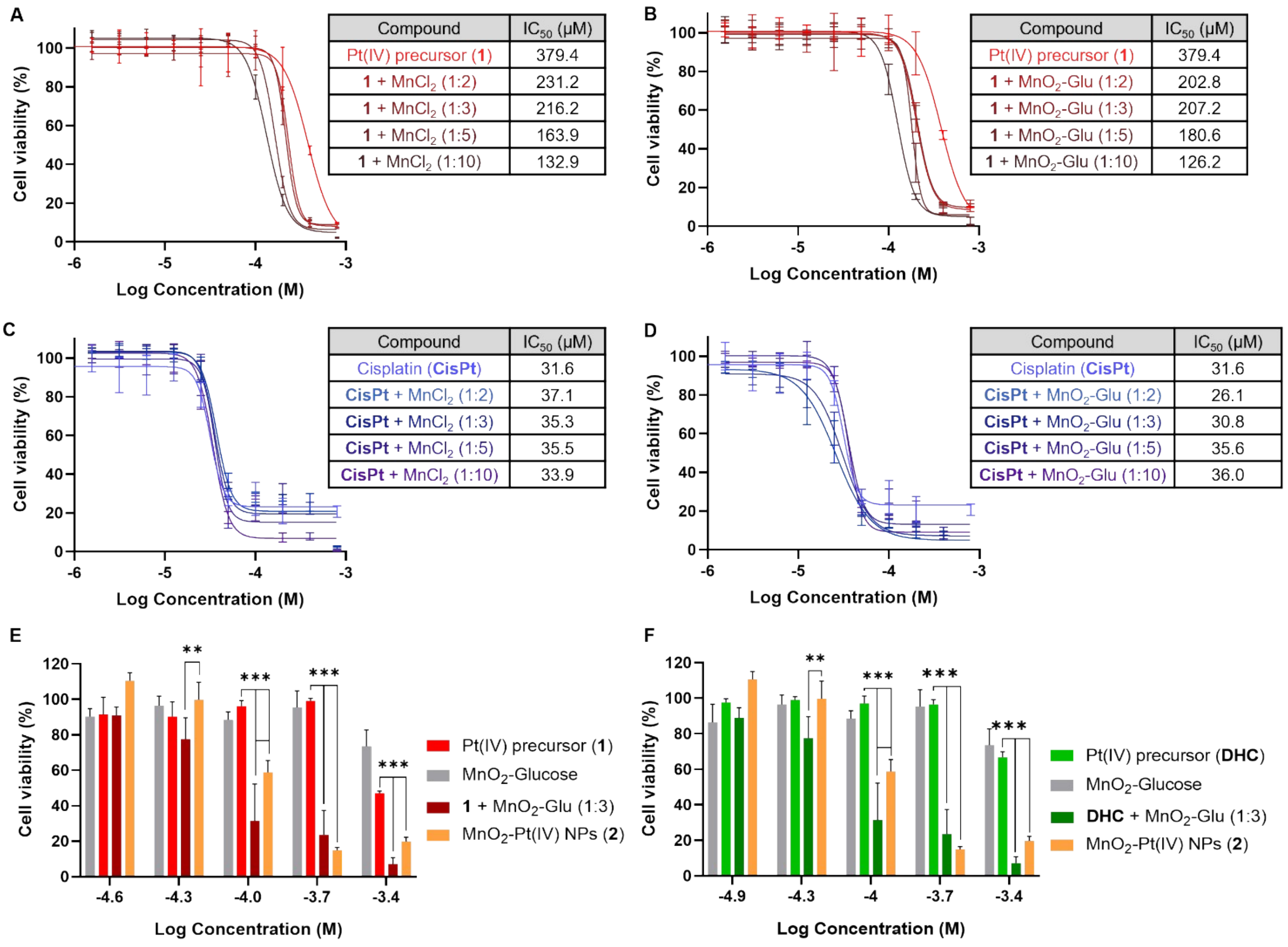

Figure S7. Cell viability studies employing 2D models of A549 cells after 48 h of treatment. Concentration refers to Pt concentration in all conditions, except for MnO_2-Glu NPs and $MnCl_2$, where Mn concentration is shown. Comparison of cell viability of Pt(IV) prodrug **1** after addition of **A)** $MnCl_2$ salts and **B)** MnO_2-Glucose nanoparticles (Mn concentration equivalent to 2, 3, 5 and 10 times the Pt concentration) and corresponding calculated IC_{50} values. Comparison of cell viability of cisplatin after addition of **A)** $MnCl_2$ salts and **B)** MnO_2-Glucose nanoparticles (Mn concentration equivalent to 2, 3, 5 and 10 times the Pt concentration) and corresponding calculated IC_{50} values. **E** and **F)** Comparison of cell viability of Pt(IV) prodrugs **1** and oxoplatin (DHC) after addition of MnO_2-Glucose nanoparticles (Mn/Pt = 3). **$p<0.0034$, ***$p<0.001$ (two-way ANOVA).

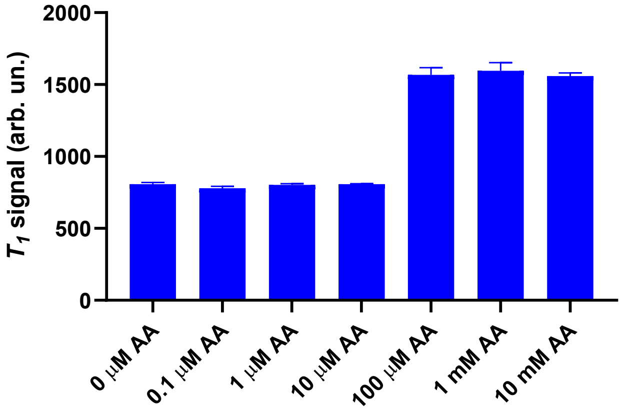

Figure S8. MR signals of MnO_2-Pt(IV) NPs in the presence of different concentrations of AA (0-10 mM) at pH=7.4, at 3 T.

Table S2. ICP-MS results from the analysis of Pt and Mn content of DNA samples extracted from cell incubated in media or in the presence of the NPs.

	DNA measure / ng	Pt / ppm	Mn / ppm
**Media control**	168.0 ± 53.7	<LLOD	0.6 ± 0.2
**NPs**	103.3 ± 38.5	12.4 ± 2.6	1.3 ± 0.7

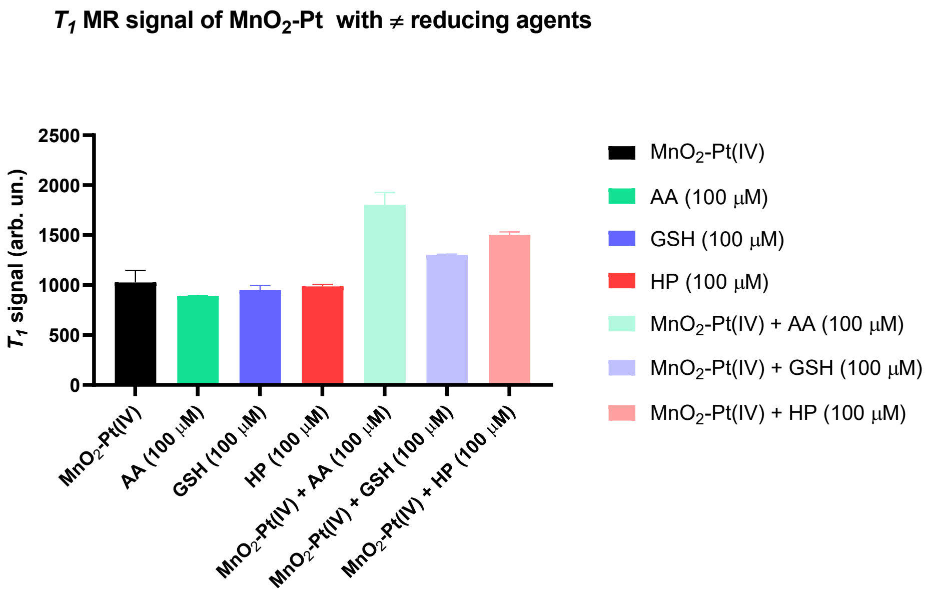

Figure S9. MR signals of MnO_2-Pt(IV) NPs in the presence of 100 μM of different reducing agents: ascorbic acid (AA), glutathione (GSH) and hydrogen peroxide (HP) at pH=7.4, at 3 T.

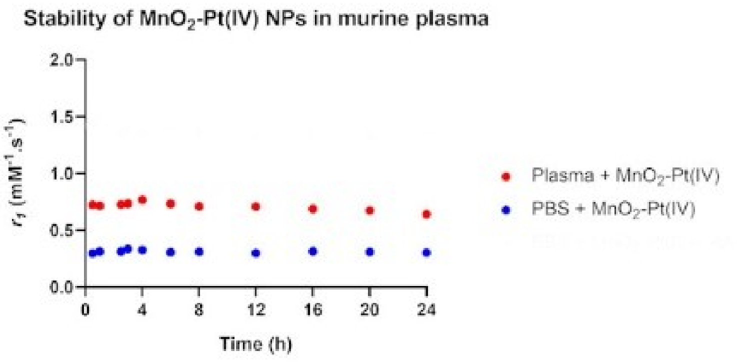

Figure S10. Relaxometry measurements of MnO_2-Pt nanoparticles in PBS or murine plasma at different time points, at 9.4 T.

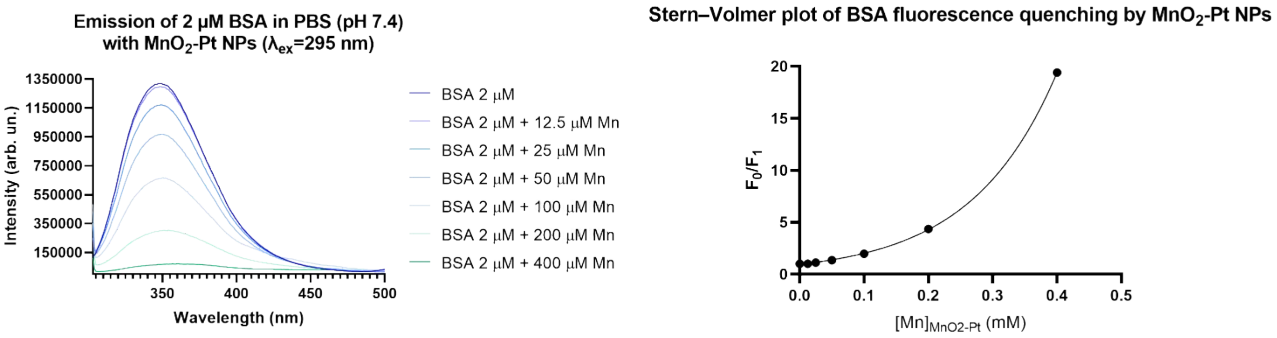

Figure S11. Left: Emission spectra of 2 μm BSA in PBS (pH=7.4) with varying concentration of MnO_2-Pt nanoparticles at λ_{ex}=295 nm. Right: Stern-Volmer plot relating [MnO_2-Pt nanoparticles] added *versus* F_0/F_1, where F_0 is the fluorescence intensity of BSA (2 μM) in PBS (pH=7.4) without quenching agent and F_1 is the fluorescence intensity of samples with MnO_2-Pt NPs added, showing dynamic quenching, indicative of dynamic binding, of MnO_2-Pt(IV) NPs to BSA.

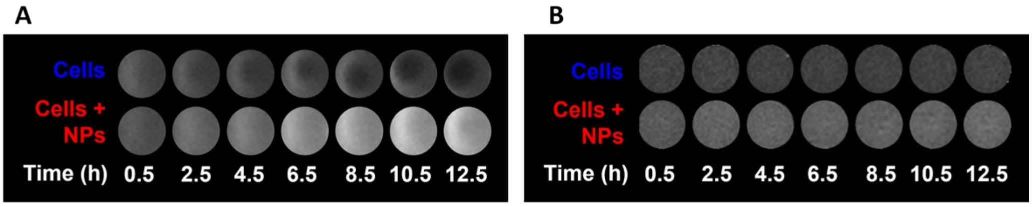

Figure S12. Representative MR phantom images of **A)** 2D and **B)** 3D A549 cells treated with MnO_2-Pt NPs (637 µM and 500 µM of Mn, respectively) over time, at 3.0 T.

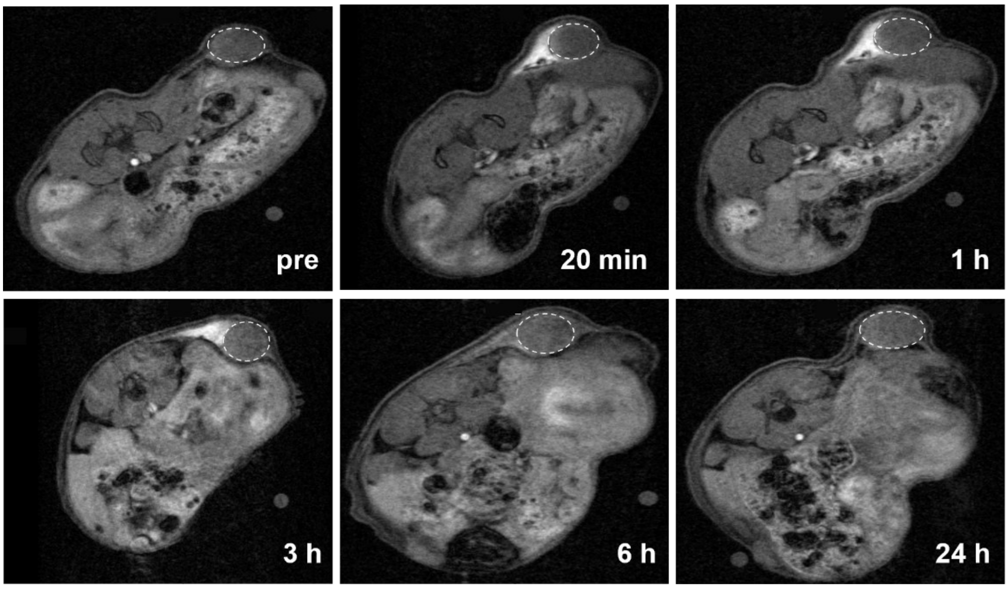

Figure S13. T_1-weighted axial MR images of tumour-bearing Balb/c nude mice before (pre) and 20 min, 1 h, 3 h, 6 h and 24 h after intratumoral injection of $MnCl_2$, acquired using a 9.4 T MR scanner. White circles highlight tumour sites.

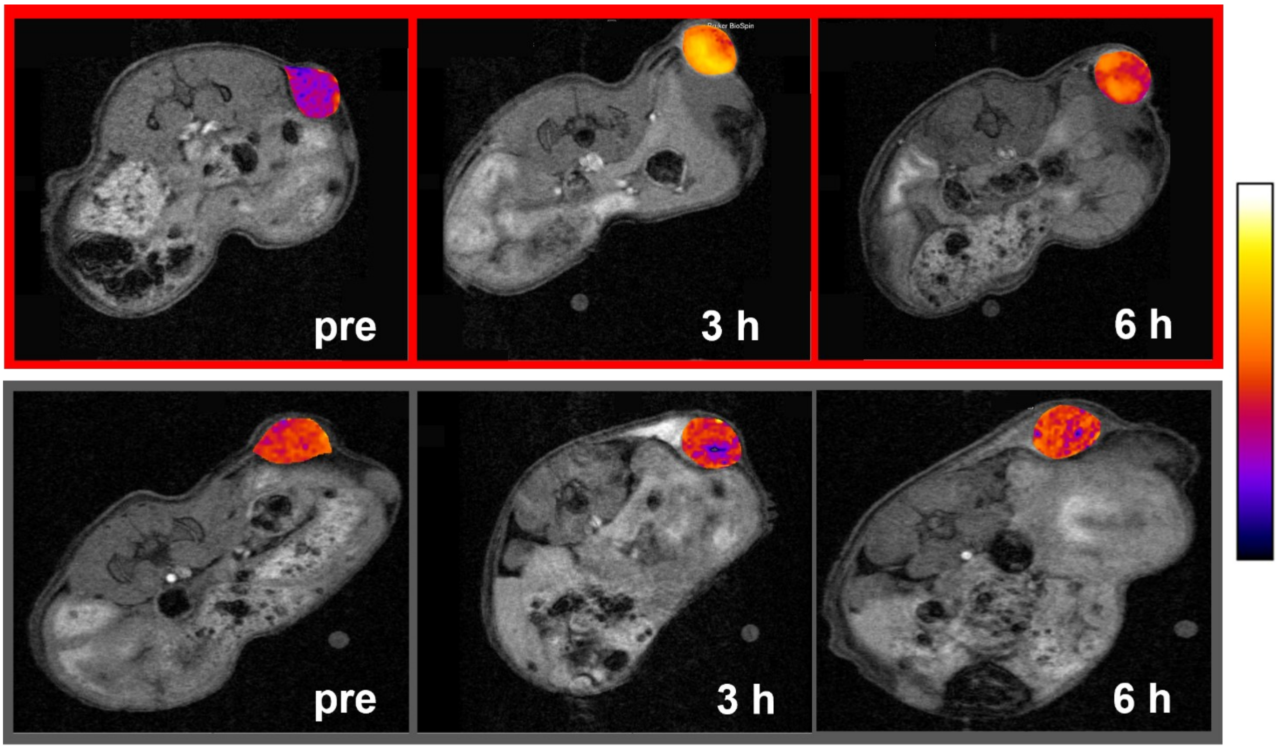

Figure S14. T_1-weighted axial MR images of tumour-bearing Balb/c nude mice before (pre), 3 h and 6 h after intratumoral injection of MnO_2-Pt(IV) NPs (above) $MnCl_2$ (below), overlapped with T_1 maps of tumours, acquired using a 9.4 T MR scanner.

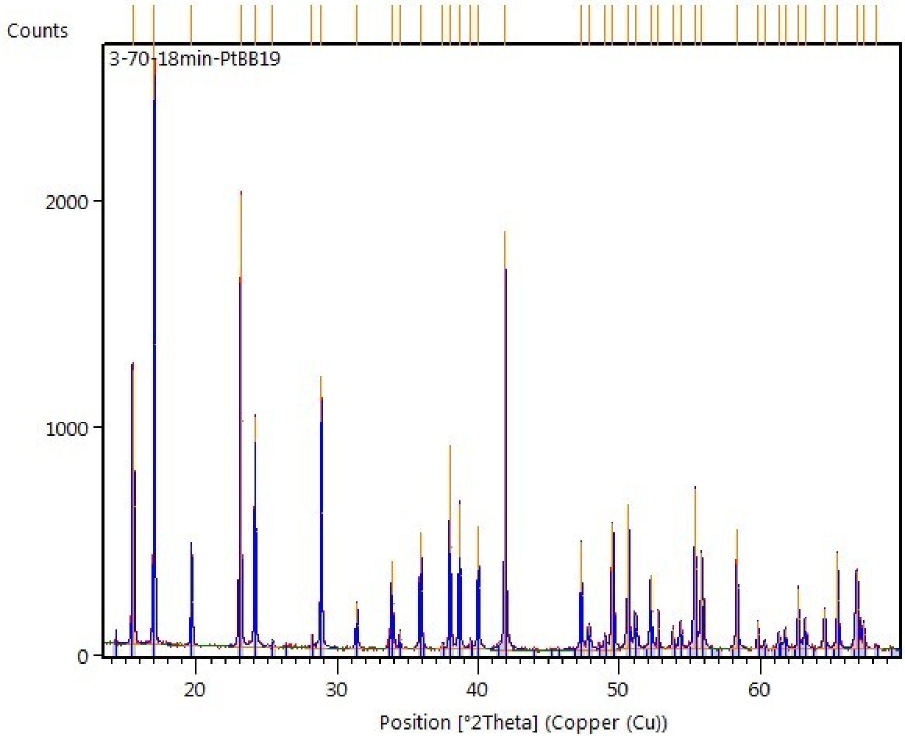

Figure S15. XRD spectrum of Pt(IV) prodrug oxoplatin (*cis, cis, trans*-Diamminedichlorodihydroxyplatinum(IV)).
